# Editors’ Note

**DOI:** 10.5195/ijt.2021.6381

**Published:** 2021-06-22

**Authors:** Ellen R. Cohn, Jana Cason

The *International Journal of Telerehabilitation* (IJT) is a biannual journal dedicated to advancing telerehabilitation by disseminating information about current research and practices. This open-source journal is indexed by PubMed and Scopus. IJT requires no readership or author fees.

## AN UPDATED JOURNAL FORMAT

The website for the International Journal of Telerehabilitation (IJT) was recently upgraded to OJS 3 which offers significant enhancements over the previous version of the software, especially for accessibility. The conversion features an updated look and feel and improved functionality that will benefit the journal and its work. We are grateful to Michael Johnston who created an updated banner graphic and a new journal format, and Vanessa Gabler, who spear-headed the OJS3 upgrade.

## ISSUE OVERVIEW

This issue was produced during an historic time. The issue's development paradoxically spanned the darkest days of the 2020 COVID-19 pandemic both in the United States and around the world, as well as the miraculously rapid development and distribution of life preserving vaccines. Yet, as we write this, our hearts hurt for our authors and readership whose locales and families are still severely challenged by the virus. We posit that both telerehabilitation and our larger society will forever be changed.

Despite the difficult circumstances and the need for most to work remotely, submissions to the journal increased dramatically during the pandemic, hence the current larger than typical issue. COVID19 apparently turned on the spigot of telerehabilitation, full force! Prospective authors and experienced reviewers met the challenge; the latter generously completed extra reviews.

The current issue of the multi-disciplinary *International Journal of Telerehabilitation* (IJT) features impactful research across rehabilitation disciplines. Among the curated articles, several detail how telerehabilitation is strategically deployed in underserved regions. Essential research on group-based telerehabilitation is presented as the lead article.

## ACKNOWLEDGEMENTS

A portion of a prior Editors' note^1^ is as relevant for this issue as it was for the previous issue:

We cannot over-state the contributions that volunteer reviewers make to a journal, its authors, and to current and future readers. Discerning reviewers are essential to the quality control of every journal. The purpose of an IJT review is not just to advise the editors on whether to publish an article. Most importantly, reviewers dig deep inside an article and if needed, advise authors how to elevate the quality of their work. We are most grateful to an assiduous group of new and returning anonymous reviewers, about whose efforts our authors routinely offer appreciation. One experienced scholar recently wrote: “Again, we want to pass on our sincere thank you for considering this submission and what a reviewer this is! They are exceptional and have provided great feedback.”

Sections Editor, William E. Janes, OTD, MSCI, OTR/L continues to be a very impactful reviewer who renders supports to many IJT authors.

Thank you also to the publisher of IJT, the University Library System, University of Pittsburgh.^2^ We applaud the expertise and the professionalism of Vanessa Gabler, Electronic Publications Manager, Office of Scholarly Communication and Publishing University Library System, University of Pittsburgh, for her long-term work with IJT.

IJT is currently sponsored by the Rehabilitation Engineering Research Center on Information and Communication Technology Access at the University of Pittsburgh. The RERC is funded by the National Institute on Disability, Independent Living, and Rehabilitation Research (NIDILRR). Volumes 1–7 were sponsored by the Rehabilitation Engineering Research Center (RERC) on Telerehabilitation at the University of Pittsburgh.

## CALL FOR SUBMISSIONS

We cordially invite submissions to the Fall 2021 issue beginning on July 1, 2021. IJT accepts original research, case studies, viewpoints, technology reviews, book reviews, and country reports that detail the status of telerehabilitation. References should adhere to the conventions of APA version 7.

Sincerely,

Ellen R. Cohn, PhD, CCC-SLP, ASHA-F, IJT Editor

Jana Cason, DHSc, OTR/L, FAOTA, Senior Associate Editor

## Footnotes

1 Vol. 12, No. 1, Fall 2020

2 https://www.library.pitt.edu/e-journals

